# Obstetrics, Gynecology and Pelvic Surgery

**Published:** 1896-03

**Authors:** William Warren Potter

**Affiliations:** Buffalo, N. Y., Examiner in obstetrics, New York State Medical Examining and Licensing Board


### OBSTETRICS, GYNECOLOGY AND PELVIC SURGERY.

CONDUCTED BY WILLIAM WARREN POTTER, M. D., Buffalo, N. Y.,
Examiner in obstetrics, New York State Medical Examining and Licensing Board.

### ECTOPIC GESTATION OCCURRING TWICE IN SAME PATIENT; TWO OPERATIONS; RECOVERY.

Ross, J. F. W., of Toronto, (*Am. Jour. Obstetrics*, February, 1896,) reports a case of his own as follows:

Mrs. H., aged 24 years; no children, though had miscarried. March 20, 1891, became "unwell" and so continued for seven weeks; then flowing ceased, but commenced again June 9th, when she was seized with pains similar to labor; no other symptoms of pregnancy. June 30th, she walked to the hospital. Two days later she entered for operation. Abdomen was filled with liquid blood, grumous in appearance, and though she had been walking around the day before operation, yet, in the opinion of the operator, the blood had been in the abdomen several days. She made an excellent recovery.

October 10, 1895, Dr. Ross was summoned by Dr. Noble to see same patient described above. He saw her within twenty minutes ; found her collapsed, pale, with all appearance of internal hemorrhage. Patient had gone two weeks over her period. Under anesthesia Ross felt blood clots in Douglas's pouch break down under the examining finger. A small mass was felt to the left of the uterus. She was removed to the hospital and within an hour after first seeing her Ross operated. Blood gushed out on section ; abdomen full of blood ; enormous clots removed. An extrauterine pregnancy was found at the fimbriated end of the left tube. The point of rupture could be seen. Patient made an uninterrupted recovery.

Ross publishes a table of five similar cases—all he has been able to find—namely, one by Lawson Tait, one by Veit, J., one by Olshausen, one by Charles A. L. Reed, and this one, his own. Other cases have been reported, but these are all in which he deems the diagnosis sufficiently well established for acceptance.

---

### USE AND ABUSE OF THE UTERINE CURETTE.

Dorsett, W. B., of St. Louis, (*American Practitioner and News*,) read a paper before the American Association of Obstetricians and Gynecologists, at its eighth annual meeting, with the above title. The more the author had used and seen used the uterine curette, the more he was impressed with the following ideas: that when used, the selection as to the shape and form of the instrument in a given case is not always a wise one; that a proper knowledge of its use should be obtained before trying to use it; that it is not a cure-all. Its use should be only in conjunction with other treatment. He looks upon the blunt curette (as sold in the shops) as a dangerous instrument. The instrument with a sharp-cutting edge, properly constructed, is a most useful one, and in the treatment of intrauterine inflammatory conditions is the *sine qua non* of success. In order to secure a good scraping instrument the sharp edge should stand at an angle of 60° to the shaft or handle. A greater angle will not scrape thoroughly, and a lesser angle is liable to incise the uterine wall unless used with a great deal of care. Cases of perforation of the uterine wall are on record, and he thinks they are due to want of care in the selection of the proper instrument. The dull or blunt curette should never under any circumstances be used.

---

### CONSTIPATION IN WOMEN.

Holmes (*Southern Medical Journal—Brooklyn Medical Journal*,) says a very frequent cause of disease in women is constipation. It is remarkable how careless many women are in this respect. The mother should educate the girl from infancy that it is just as important to keep her bowels open as to sleep and eat. We find girls frequently going from three to five days, in some instances longer, without a movement from the bowels. Not only do they have from this a poisoning of the system from absorption of the liquid and gaseous contents of the bowels, the ptomaines or poisons developed in them from fermentation, producing extremely depressing effects on the nervous system, with great derangement of the stomach and assimilative organs, as shown in the pale faces, debility, neuralgia, headache, and a general feeling of exhaustion ; but we get, in addition, from impacted feces in the rectum, uterine displacement, with its consequent disturbances in the pelvic circulation and with its general reflex neuroses. It is a well-known fact to gynecologists that the left ovary is oftener diseased than the right one. The left ovarian vein has no valve, and a slight pressure upon it prevents its emptying. Doubtless the pressure of a loaded rectum in this event is a prolific cause of disease of the ovary, especially the left.

---

### TACHYCARDIA AT THE MENOPAUSE.

Baldwin (*Brooklyn Medical Journal*,) says tachycardia may occur at any period of life. It is rare, however, before puberty and after fifty years of age. It is most often seen during the menopause. Early menopause predisposes to it, and especially if prematurely induced by surgical operation or disease. Its etiology is obscure. Stimulation of the sympathetic, hyperplasia of the stroma in sexual organs, the formation of scar tissue at the seat of operation in surgical cases, are some of the suggested causes. It may be paroxysmal or continuous ; it may last for a few moments, hours, days or years. It may be precipitated by use of alcoholic liquors, strong coffee, anxiety or sleeplessness. The immediate results of a rapid heart are various ; some experience grave apprehension and alarm, some are bathed in perspiration, some have epistaxis. The attack may disappear slowly or quickly. It may prove fatal. The heart always has a reserve power that will enable it to make twice its normal pulsations for a certain time, but when this reserve is exhausted, then comes the danger, for its nutrition has become seriously impaired.

Treatment: Opium and digitalis stand at the head, but often prove worthless. Combined and judiciously used they are often successful. Nitro-glycerine, in combination with digitalis, is valuable. Galvanism has been the most effective agent, but was limited to the time of giving it. One sponge over the heart, the other over the left ear, in severe cases can render valuable service. General hygienic care advised, cool morning baths, the relief of indigestion and constipation; for the former, resorcin; for the latter, compound aloin pill.

---

### GONORRHEA IN WOMEN.

THE course of gonorrhea in the female (*Philadelphia Polyclinic*,) is a rapidly progressing one, the infection quickly spreading from the vagina to the endometrium and then to the Fallopian tubes. Appreciating this fact and also that the danger to the patient increases in an alarming ratio with the progress of the infection, the aim of the physician would naturally be to prevent the spread and to destroy the disease in the parts already infected. When the disease is limited to the vagina, Dr. Talley recommends the daily washing of the mucous membrane with solution of *mercuric chlorid* (1-2000) and the filling of the upper part of the vagina with dry powdered *tannic acid.* A dry cotton tampon is then introduced to secure the retention of the powder. The cervix must be carefully watched for evidence of the infection of the endometrium, which will be shown by a red or granular condition of the external os and the flowing of mucus from the cervical canal. Should this be noticed, irrigation of the uterus with one or two gallons of a mild alkaline antiseptic solution at a temperature of 110° F., followed by the injection of equal parts of Churchill's iodin and carbolic acid, will, in the majority of cases, prevent the spread to the tubes.

---

### A CLINICAL CONTRIBUTION TO THE STUDY OF THE LATERAL DISPLACEMENTS OF THE UTERUS.

ILL, EDWARD J., of Newark, read a paper on this subject before the American Association of Obstetricians and Gynecologists at Chicago. After reviewing the literature, he spoke of the importance of this abnormal condition, and thought it had been generally overlooked, the patient's symptoms being attributed to other ailments. He had collected from his last year's office case-book all cases of lateral displacements except such as had presented tumors. He showed that in 14.2 per cent. there had been lateral displacement.

He drew special attention to those cases which he considered to be congenital and where the pain was referred to the elongated broad ligament. The symptoms began early in the patient's sexual life, in severer cases progressing gradually to complete invalidism. He then related *in extenso* several histories. He described a typical case. The nonoperative treatment consisted in endeavoring to elongate the shortened ligament by the use of dry wool or oakum tampons pushed between the cervix and the ilium on the side of the shortened ligament, keeping the tampon in place by a second and third one. All this was to be retained for forty-eight hours. A hot douche, with the patient on her knees and elbows, twice daily, when the tampons were *in situ*, was also ordered. He related two cases of extreme suffering where total extirpation of the uterus, tubes, ovaries and broad ligament had been deemed advisable after years of unsuccessful treatment, in both of which the patients had been much relieved of their suffering by this method.

---

### SHOULD INTRAUTERINE INJECTIONS OF GLYCERINE BE USED FOR THE INDUCTION OF LABOR?

HYPES, in a paper read at the annual meeting of the American Association of Obstetricians and Gynecologists, at Chicago, September, 1895, reports a case (*American Journal of Obstetrics*, December, 1895,) where glycerine was used to induce labor in a healthy young woman, 23 years old, who had no kidney lesion prior to the use of Pelzers' method, but who died of acute nephritis after its use. He then enters into a discussion of a number of other cases which have appeared, treated by this method, and of the effects upon the system produced by the absorption of a large amount of glycerine. Glycerine in these amounts (2½ ozs.) appears to alter the composition of the blood with the production of methemoglobin, and of disintegration of the red disks. Hematuria appears and the blood remains fluid after death. Evidences of intense congestion of various organs appear—kidneys, liver, stomach, bowels, brain and nervous system.

Dr. Hypes comes to the following conclusions: Intrauterine injections (of glycerine) are often inefficient, especially so in doses under fifty cubic centimeters. They are liable to be followed by all the ill effects—shock, air embolism, thrombosis, metritis and sepsis—of other intrauterine douches which have been used and abandoned during the past century. They may and sometimes do produce glycerine poisoning—*i. e.*, decomposition of the blood corpuscles—resulting in diseases of various organs, but more especially in nephritis with hemoglobinuria. This method takes no consideration of the life of the child, and hence results in great mortality. Its use should be abandoned or the dosage reduced, especially in subjects with prior existing kidney affections.

---

### THE INDICATIONS FOR OPERATION IN PUERPERAL SEPSIS.

McMurtry, Louisville, Ky., read a paper with the above title at the Chicago meeting of the American Association of Obstetricians and Gynecologists, in which, *inter alia*, he said the efficiency of aseptic methods in preventing infection during the puerperium has been demonstrated by the recorded results of maternity hospitals. Since operative surgery a few years since disclosed the various lesions of pelvic disease, it has been known that pregnancy and the puerperal state may be complicated by preëxisting inflammatory diseases of the uterine appendages, tumors and septic accumulations inside the pelvis; chronic and circumscribed disease of this character may be converted into acute and diffuse inflammatory conditions by the trauma of labor. Puerperal sepsis may in this way be the result of preëxisting disease. This class of cases must necessarily be small, since women thus diseased are generally sterile. That such cases necessarily come within the scope of operative treatment will be generally conceded. The author then considers the indications and guides for operative interference. For practical purposes he divides puerperal sepsis into two general divisions:

(1) Those cases wherein systemic infection is marked and prominent with comparatively insignificant local manifestations; and (2) those wherein the local inflammatory lesions are conspicuous and general systemic infection less marked and secondary. In the first division, by the time the diagnosis is made, the mischief is done and nothing avails; in the second division, when the lesions are demonstrable to the skilled touch and local signs of known value, together with general symptoms of recognised significance are present, they form the basis of decisive action. The author closes his paper by deprecating empirical operations, such as hysterectomy, in the class of puerperal cases where the local symptoms are those of diffuse peritonitis without localisation of lesions.

